# Detection of polyomavirus microRNA-5p expression in saliva shortly after kidney transplantation

**DOI:** 10.1080/20002297.2021.1898838

**Published:** 2021-03-12

**Authors:** Ana Carolina Mamana, Maria Stincarelli, Dmitry José De Santana Sarmento, Alexandre Mendes Batista, Tânia Regina Tozetto-Mendoza, Marina Gallottini, José Osmar Medina De Abreu Pestana, Paulo Henrique Braz-Silva, Simone Giannecchini

**Affiliations:** a Laboratory of Virology, Institute of Tropical Medicine of São Paulo, School of Medicine, University of São Paulo, São Paulo, Brazil; b Department of Experimental and Clinical Medicine, University of Florence, Florence, Italy; c Department of Stomatology, School of Dentistry, University of São Paulo, São Paulo, Brazil; d Division of Renal Transplantation, Kidney and Hypertension Hospital, Federal University of São Paulo, São Paulo, Brazil

**Keywords:** Polyomavirus, polyomavirus microRNA-5p, saliva, renal transplantation, polyomavirus-associated diseases

## Abstract

**Background:** MicroRNAs (miRNAs) of polyomavirus (PyV) are present in several biological fluids and are suggested to be relevant viral factors for monitoring its persistence.

**Aim:** To evaluate the effect of an immunosuppressive regimen on the status of PyV-miRNA-5p in the oral cavity.

**Materials and Methods:** The JCPyV, BKPyV, MCPyV miRNA-5p were investigated in paired saliva and plasma samples obtained from 23 patients before and shortly after renal-transplantation by using real-time RT-PCR.

**Results:** Overall, within a short-time after transplantation, patients exhibited decreased numbers of leukocyte and lymphocyte as well as low levels of creatinine. During the clinical management of the patients, a significant amount of saliva samples were positive for JCPyV and BKPyV miRNA-5p (range: 26%-91%) compared to paired plasma samples (range: 9%-35%). Among the two polyomaviruses showing positive expression of miRNA-5p, BKPyV presented the highest positivity in saliva (91%) and MCPyV-miRNA-5p was constantly negative in both saliva and plasma samples. Compared to the time before transplantation, a significant reduction in the expression of JCPyV-miRNA-5p was observed in saliva samples obtained after transplantation.

**Conclusions:** Altogether, these data suggest that additional investigations of polyomavirus miRNA-5p in saliva should be performed shortly after renal-transplantation to evaluate the potential role in early viral reactivation.

## Introduction

Polyomaviruses (PyVs) are non-enveloped, small icosahedral viruses with a closed circular, super coiled, double-stranded DNA genome [1]. After the discovery of the first isolates of human polyomavirus in 1971, JC (JCPyV) and BK (BKPyV), recent advances in DNA sequencing technologies have identified up to 14 polyomaviruses infecting humans [2,3]. In addition, simian virus 40 (SV40) and lymphotropic polyomavirus DNA, two non-human primate polyomaviruses, have been detected in humans [4,5]. Primary polyomavirus infections predominantly occur in early childhood, with the main routes of transmission being oral-fecal or respiratory. The seroprevalence rates for polyomaviruses in healthy populations range from 23% to 98% [6]. In this context, polyomaviruses causing long-term persistent infections are a serious concern in immunocompromised patients [1,7].

Among the human polyomavirus-associated diseases, the two major complications associated with BKPyV mainly affect kidney transplant patients (nephropathy) [8–10] and allogeneic hematopoietic stem cell transplant patients (hemorrhagic cystitis) [11–13], only sporadically occurring in patients with other solid organ transplantation or other immune deficiencies [14–16]. Although the mechanism underlying the reactivation of polyomaviruses is not yet well known, it seems that variations in their non-coding control region (NCCR) are associated with high viral replication activity, which is implicated in the development of polyomavirus-associated diseases [2,17].

Because infections caused by persistent viruses, such as polyomavirus, continue to challenge the clinical management of transplant recipients, it is important to monitor virus reactivation in transplant patients [15,18]. In this context, in the absence of therapies, screening kidney transplant patients for BKPyV and JCPyV replication in urine and blood is a key recommendation when changing the immunosuppression therapy in patients with BKPyV and JCPyV viremia. In this regard, it is of great interest to perform virus detection analyses in different biological fluids (i.e. plasma, serum, cerebrospinal fluid, urine) from individuals with chronic kidney failure or from kidney transplant patients in order to assess viral replication and risk for disease [19–23].

An increasing evidence has suggested that there is a connection between human polyomaviruses and the oral compartment [1,24–28], meaning that saliva can be used as an additional biological fluid to assess the polyomavirus status. Therefore, saliva has gained an increased interest as a non-invasive screening approach to facilitate detection of new cases and monitor previously known cases [19]. Polyomavirus-encoded microRNA (miRNA-3p and −5p), which down-regulates early viral genes (mainly polyomavirus-miRNA-5p) and target host factors (mainly polyomavirus-miRNA-3p) to aid in the escape of polyomaviruses from immune elimination [29–31].

Polyomavirus-miRNAs have been detected in numerous biological fluids, such as urine, plasma, serum, cerebrospinal fluid (CSF) and saliva, and their presence has been associated with viral reactivation status and polyomavirus-associated disease outcome in natalizumab-treated multiple sclerosis patients, transplant patients with low or high immunosuppressive activity, HIV-infected patients and healthy individuals [32–38]. Polyomavirus -miRNAs have also been detected in low-replicative conditions, in which viral DNA is not present in the biological fluid [34]. Additionally, polyomavirus-miRNAs are more highly expressed in polyomaviruses carrying the NCCR archetype than in those carrying the mutated form of NCCR [39,40], meaning that there is a direct correlation with polyomavirus pathology and an indirect correlation with viral load [34,35,41].

The purpose of this study was to evaluate the effect of an immunosuppressive regimen on the status of polyomavirus -miRNA-5p in the oral cavity. In order to do this, the polyomavirus-miRNA-5p circulating in the saliva of renal transplant patients was investigated to assess the effectiveness of this non-invasive approach in screening the polyomavirus status in such patients. This was relevant because polyomavirus-miRNA-5p plays a role in controlling virus replication, meaning that a potential reduction in the miRNA-5p expression can be a risk factor for viral reactivation. Therefore, the polyomavirus-miRNA-5p was specifically investigated in saliva samples obtained from 23 renal transplant patients before and after transplantation. Paired plasma samples obtained from the same subjects were used as controls.

## Materials and methods

### Samples

This is a cohort study conducted in a renal transplant unit of the Federal University of São Paulo Kidney and Hypertension Hospital, São Paulo, Brazil. Patients older than 18 years who underwent single kidney transplantation were initially included. Exclusion criteria were kidney transplants associated with another organ, immunosuppressive therapy before initiation of the study, and HIV positivity. This study was approved by the Research Ethics Committees of the Kidney and Hypertension Hospital and of the Osvaldo Ramos Foundation, according to protocol number 2,362,239, and by the University of São Paulo School of Dentistry, according to protocol number 1,824,857, following the ethical standards set by the Declaration of Helsinki. All the participants signed a free informed consent form before being examined in two different periods, always by the same examiner, as follows: the first examination within 24 hours before renal transplantation and the second 60 days after the surgery. All clinical data in the patients’ charts, including laboratory test values, were assessed. For intraoral examination, the same experienced dentist used the decayed, missing and filled teeth index (DTMF) and community periodontal index of treatment needs (CPITN). Collection of saliva and blood samples was also performed for molecular analysis. Whole saliva samples were obtained without previous stimulation, that is, by asking the patient to spit into a sterile container. Blood samples were collected at the same moment as the saliva collection. After collection and identification, the blood and saliva samples were centrifuged at 800 rpm in a conical tube, and then 500 μL aliquots of the supernatant of each sample obtained after centrifugation were placed into cryotubes for storage in a freezer at −80°C until laboratory analysis.

### Quantification of polyomavirus miRNA-5p

Total RNA was isolated from 250 µl of each saliva and plasma samples, which had been previously centrifuged at 14,000 g for 20 minutes, by using a kit for extraction of circulating and exosomal RNA (Norgen) according to the manufacturer’s protocols. Expression of PyV-miRNA-5p was specifically analysed and quantified for JCPyV (jcv-miR-J1-5p; Assay ID 007464_mat), BKPyV (bkv-miR-B1-5p; Assay ID 007796_mat) and MCPyV (mcv-miR-M1-5p; Assay ID 006356_mat) by using miR-5p quantitative stem-loop RT-PCR miRNA assays (Life Technologies, Foster City, CA, USA) according to the manufacturer’s protocol. Each reaction was carried out by using 10 ng of RNA, which included a negative control (no template) and synthesized oligonucleotides as standards (diluted to contain 10^1^–10^6^ copies). The reverse transcription was carried out at 42°C for 30 minutes and terminated by a further incubation at 85°C for 5 minutes. Real-time PCR amplification was performed by using a thermal profile beginning at 95°C for 10 minutes, followed by 40 cycles at 95°C for 15 seconds and at 60°C for 60 seconds. The lower limit of detection was 10 copies of viral miRNA-5p *per* reaction based on the amplification of synthesized oligonucleotides standard and testing samples. As reported in a previous study [38], the assay specificity for JCPyV and BKPyV miRNA-5 showed only marginal cross-reactions in their combinations as only 15 and 25 copies, respectively, were amplified compared to an input nominal copy number concentrations of up to 10^4^ copies of polyomavirus- miRNA-5p in the synthesized oligonucleotide standards used. Moreover, spiking experiments by using synthetic unrelated miRNA spiked into saliva and plasma samples negative for polyomavirus miRNA-5p were performed without amplification of unrelated target to confirm the specificity of the assay.

### Statistical analyses

The resulting data were analysed by using chi-square and Student’s t-tests. *P*-values less than 0.05 and 0.01 were considered statistically significant.

## Results

### Demographic and clinical characteristics of the renal transplant patients

Twenty-three patients undergoing renal transplantation at the Renal Transplant Unit of the Federal University of São Paulo Kidney and Hypertension Hospital were selected for study (Table 1), being 13 (56%) females and 10 (39%) males with ages ranging from 19 to 69 years old and mean (standard deviation) age of 40.9 ± 13.6 (41.9 ± 10.9 and 39.7 ± 16.7 for females and males, respectively). None of the patients reported to be alcoholic and only one had a positive smoking status. Among the patients, 17 (74%) were on hemodialysis and three (13%) on peritoneal dialysis for a period from 7 to 75 months. Dialysis treatment was not performed for three (13%) patients as they had been submitted to transplant without undergoing renal replacement therapy. The patients primarily received a transplant by using the kidney from a living donor, typically a relative. Twenty-two out of 23 (96%) patients did not receive prophylactic antiviral therapy due to a serological positive status for CMV (Supplementary Table 1s), although some cases had received an organ from a CMV-positive donor. One patient who was serologically negative for CMV received a transplant from a donor who was not serologically characterized for CMV, but the recipient was not given prophylactic antiviral therapy.

**Table 1.** Demographic and clinical characteristics of study patients

Patient^a^				TYPE OF	TIME OF	TYPE OF		CMV IgG							
ID	SEX	AGE	DIALYSIS	DIALYSIS	DIALYSIS (Mo)	DONOR	RELATIONSHIP	RECEPTOR	DONOR		DISEASE		COMORBIDITIES		
1	F	32	YES	DIALYSIS	11	DEAD	none	+	+		HYPERTENSION		YES		
4	F	41	YES	HEMODIALYSIS	15	DEAD	none	+	+		ALPORT SYNDROME		NO		
6	M	42	YES	HEMODIALYSIS	58	DEAD	none	+	+		HYPERTENSION		YES		
7	M	24	YES	HEMODIALYSIS	7	ALIVE	FATHER/ MOTHER	+	+		GLOMERULONEPHRITIS		NO		
9	F	34	YES	DIALYSIS	18	ALIVE	FATHER/ MOTHER	+	+		DIABETES MELLITUS		YES		
10	M	38	YES	HEMODIALYSIS	25	ALIVE	WIFE	+	+		HYPERTENSION		YES		
11	F	39	NO	Na		ALIVE	FATHER/ MOTHER	+	+		NEPHROPATHY OF IgA		YES		
12	F	49	YES	HEMODIALYSIS	75	DEAD	none	+	+		POLYCYSTIC KIDNEY		NO		
13	F	46	YES	HEMODIALYSIS	12	ALIVE	BROTHER	+	+		GLOMERULONEPHRITIS		YES		
14	M	20	YES	HEMODIALYSIS	46	ALIVE	FATHER/ MOTHER	+	-		GESF		YES		
15	F	29	YES	DIALYSIS	6	ALIVE	FATHER/ MOTHER	+	ND		GESF		YES		
16	M	47	YES	HEMODIALYSIS	35	DEAD	none	+	+		ANTI-INFLAMMATORY DRUG USE		YES		
18	M	47	YES	HEMODIALYSIS	73	ALIVE	COUSIN	+	ND		ALPORT SYNDROME		NO		
19	M	69	YES	HEMODIALYSIS	18	DEAD	none	+	ND		HYPERTENSION		YES		
20	M	30	NO	Na		ALIVE	FATHER/ MOTHER	+	ND		ONE KIDNEY		YES		
21	F	50	YES	HEMODIALYSIS	27	ALIVE	BROTHER	+	+		UNKNOWN		YES		
23	F	66	YES	HEMODIALYSIS	15	DEAD	none	+	ND		HYPERTENSION		YES		
24	M	61	YES	HEMODIALYSIS	23	DEAD	none	-	ND		POLYCYSTIC KIDNEY		NO		
25	F	53	YES	HEMODIALYSIS	48	DEAD	none	+	ND		POLYCYSTIC KIDNEY		NO		
29	F	35	YES	HEMODIALYSIS	9	ALIVE	BROTHER	+	+		UNKNOWN		YES		
30	M	19	NO	Na		ALIVE	GRANDFATHER	+	ND		UNKNOWN		YES		
31	F	27	YES	HEMODIALYSIS	11	ALIVE	BROTHER	+	ND		GESF		YES		
32	F	44	YES	HEMODIALYSIS	22	ALIVE	FATHER/ MOTHER	+	+		UNKNOWN		YES		

^a^All patients were hospitalized in Kidney and Hypertension Hospital, São Paulo, Brazil. Na, not applicable. ND, not determined.

GESF, Glomerulonephritis focal segmental scleroderma.

**Figure 1.**
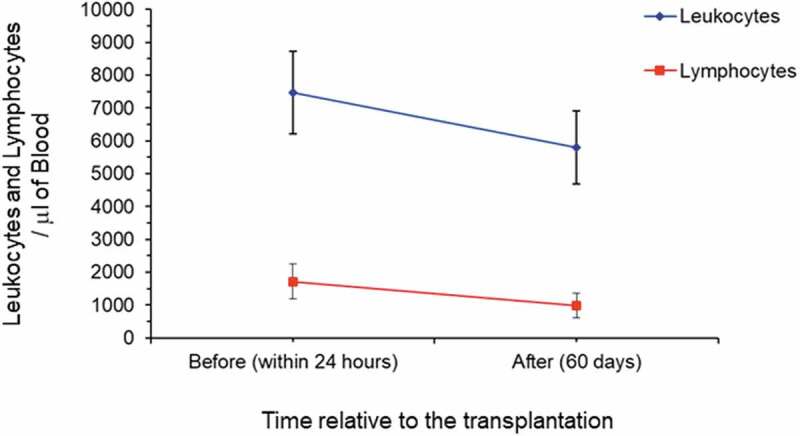
Blood cell counts in the study patients. Leukocyte and lymphocyte cell counts (µL) in the 23 patients before (within 24 hour) and after (60 days) renal transplantation. The values are given as mean ± standard deviation

Immunosuppression was induced in all patients by means of polyclonal antibodies at the time of transplantation, and soon after the surgery, they started receiving specific immunosuppressive drugs (i.e. alternative combination of tacrolimus, cyclosporine, mycophenolate sodium, azathioprine, prednisone, everolimus) (see Supplementary Table 1s). (Figure 1) shows the reduction in blood cell counts between the time before surgery (within 24 hours before transplantation, when the immunosuppressive regimen started) and 60 days after transplantation. Additionally, compared to the time before transplantation (within 24 hours), reduction in the levels of creatinine was observed 60 days after it (9.5 ± 2.7 mg/dL *versus* 1.5 ± 0.9 mg/dL, obtained before and after transplantation, respectively) (see Supplementary Table 2s). Among the 23 patients, 17 showed comorbidity with hypertension (29%) and polycystic kidney (18%), which were the main underlying diseases. Additionally, evaluation of the patients’ oral conditions showed a slightly increased number of caries and periodontitis (10 positive oral health status *versus* 15 positive oral health status) before (within 24 hours) and after (60 days) transplantation, respectively. There was a significantly high number of patients with oral lesions (candidiasis and ulcerations) after transplantation (1 positive oral lesion *versus* 7 positive oral lesions, *P = *0.012; chi-square test) (see Supplementary Table 2s). No obvious associations between demographic and clinical characteristics (i.e. age, gender, and leukocyte and lymphocyte counts) or immunosuppression treatments were observed.

### Status of polyomavirus miRNA-5p in saliva and plasma samples

**Figure 2.**
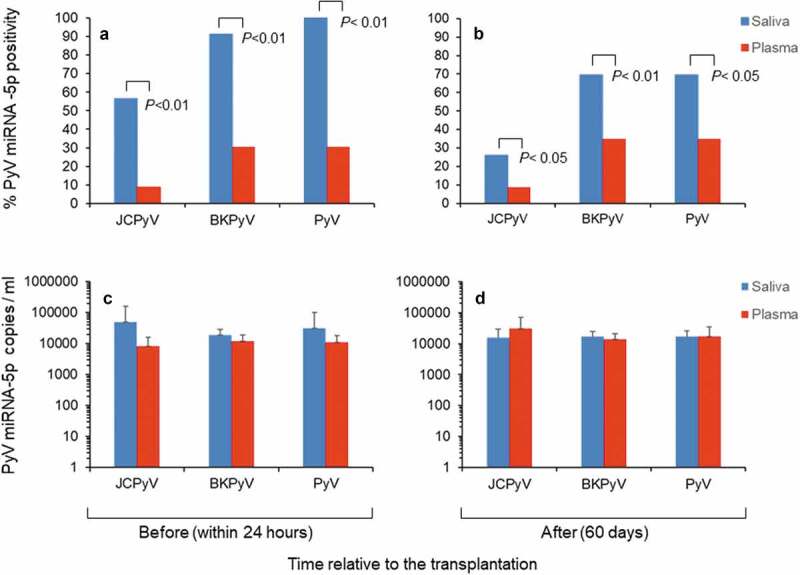
Status of polyomavirus miRNA-5p in saliva and plasma samples from the study patients. (**A-C**). Positivity for polyomavirus miRNA-5p and copy number (ml) observed in paired saliva and plasma samples before (within 24 hours) renal transplantation. (**B-D**) Positivity for polyomavirus miRNA-5p and copy number (ml) observed in paired saliva and plasma samples after (60 days) renal transplantation. In **B** and **D** the values are given as mean ± standard deviation. *P-*values (*P* < 0.01 and *P* < 0.05, chi-square test) are given

All data on the statuses of polyomavirus miRNA-5p in paired saliva and plasma samples before and after the transplantation are given in Supplementary Tables 3s and 4s. Altogether, a significant high number of saliva samples were positive, ranging from 13 (56%) to 21 (91%) before transplantation and from 6 (26%) to 16 (69%) after it for miRNA-5p regarding JCPyV and BKPyV. In the paired plasma samples, JCPyV and BKPyV miRNA-5p positivity ranged from 2 (9%) to 7 (30%) before transplantation and from 2 (9%) to 8 (35%) after it (Figure 2A and B). Among the three types of polyomavirus miRNA-5p examined, BKPyV miRNA-5p had the highest positivity (91%), whereas MCPyV miRNA-5p was constantly negative in both saliva and plasma samples. Collectively, positive saliva samples showed higher copy numbers of JCPyV-miRNA-5p and BKPyV miRNA-5 than those observed in paired plasma samples, especially before (within 24 hours) transplantation (Figure 2 C and D). Moreover, co-expression of BKPyV and JCPyV miRNA-5p was observed in the same saliva samples before and after transplantation in 48% (11/23) *versus* 26% (6/23) of the samples, respectively (Supplementary Table 2s). Conversely, double positivity was observed in plasma samples for BKPyV and JCPyV miRNA-5p in 4% (1/23) and 9% (2/23) of the samples before and after transplantation, respectively (Supplementary Table 3s).

**Figure 3.**
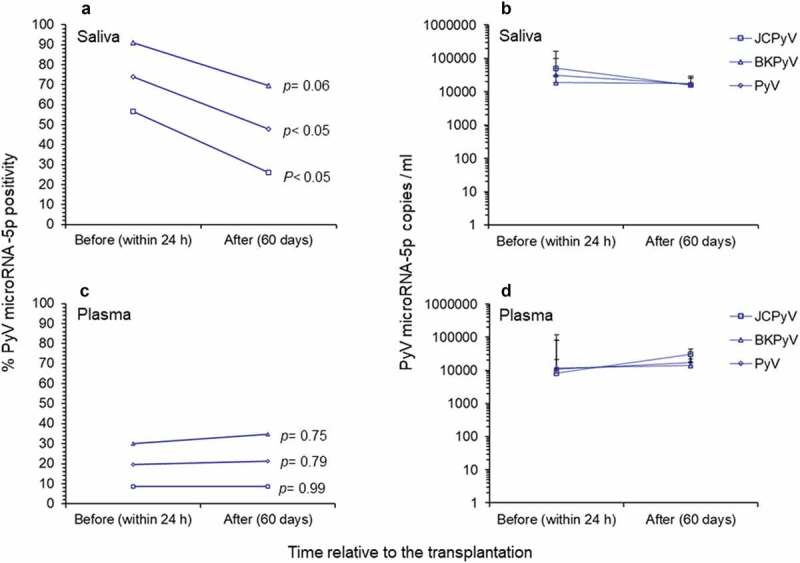
Comparison of polyomavirus microRNA-5p in study patients before and after renal transplantation. (**A-B**) Positivity for polyomavirus miRNA-5p and copy number/ml observed in saliva samples before (within 24 h) and after (60 days) renal transplantation. (**C-D**) Positivity for polyomavirus miRNA-5p and copy number/ml observed in plasma samples before (within 24 h) and after (60 days) renal transplantation. In **B** and **D** the values given are the means ± standard deviation

When the saliva samples collected before and after transplantation were compared, it was observed a significant reduction in positivity only for JCPyV miRNA-5p (*P = *0.03, chi-square test, Figure 3) was observed. Of note, the copy number of polyomavirus miRNA-5p in all positive saliva samples indicated no significant reduction in its expression before and after transplantation. Conversely, no significant reduction effect was observed in the positivity of polyomavirus miRNA-5p or in its expression in paired plasma samples before and after transplantation (Figure 3).

Finally, correlations between expression of polyomavirus miRNA-5p and presence of oral lesions were investigated. Among the seven patients who developed oral lesions (candidiasis and ulcerations) after transplantation, although not significant, four (57%) were positive for BKPyV and five (71%) for JCPyV as there was a reduction in the copy number of miRNA-5p in their saliva after transplantation.

## Discussion

In the present study, the expression of polyomavirus miRNA-5p was investigated in saliva samples collected from patients before (within 24 hours) and after (60 days) renal transplantation. Under an immunosuppressive treatment regimen, the patients showed reduction in the leukocyte and lymphocyte counts and creatinine levels 60 days after transplantation. Moreover, these patients showed a slightly increased number of caries and periodontitis after transplantation and a significantly high number of patients developed oral lesions (candidiasis and ulcerations). In this context, the status of polyomavirus miRNA-5p in saliva was significantly more positive compared to that observed in paired plasma samples both before and after transplantation, with a high number of samples positive for miRNA-5 produced by both JCPyV and BKPyV, with the latter showing the highest positivity (91%). Conversely, MCPyV-miRNA-5p was consistently negative. Moreover, the co-expression of more than one single species of miRNA-5 was observed in the same saliva samples before transplantation. Interestingly, a significant reduction in the positivity for JCPyV-miRNA-5p was observed in the comparison between the saliva samples collected before and after transplantation.

To date, it is well established that post-transplantation patients in the long term are at risk for several diseases associated with polyomavirus reactivation in different tissues, including the oral cavity [7,10,42]. Among these diseases, it seems that polyomavirus-associated renal disease primarily occurs in immunosuppressed renal transplant patients and can be attributed to several factors, including donor-organ and recipient determinants, immunosuppressive drug treatment and other modulating factors present after transplantation [10,43–45]. Moreover, with the emergence of changes in the NCCR, which modulates the rate of viral replication, and genetic variability in BKPyV may also play a relevant role [15].

BKPyV-miRNAs present in the blood circulation have been considered as additional virological factors to be monitored for risk of polyomavirus-associated diseases, especially in immunocompromised patients. In this effort, BKPyV-miRNAs have been reported to be highly expressed in urine samples from patients with polyomavirus-associated nephropathy after renal transplantation [35,37,46–49]. Although these studies reported that the expression of BKPyV-miRNAs is directly correlated with BKPyV viral load, in another one evaluating patients with multiple sclerosis on natalizumab therapy, an indirect correlation was observed [40]. Notably, in the latter study, the low expression of BKPyV-miRNAs was associated with changes in the NCCR [40]. It was recently reported that BKPyV-miRNA-3p is highly expressed in renal transplant patients during viremia compared to miRNA-5p, thus suggesting that the immune evasion functions (mainly exerted by polyomavirus-miRNA-3p) overcome the negative auto-regulatory control (mainly exerted by polyomavirus-miRNA-5p) in the active viral replication [46]. In the present study, considering the significantly high positivity for polyomavirus-miRNA-5p in saliva compared to that in paired plasma samples, it is likely that saliva reflects the state of the virus in the oral cavity, where the non-pathogenic transmissible form of polyomavirus predominantly persists [24]. Thus, although it was observed a significant reduction in miRNA-5p only for JCPyV was observed, the status of polyomavirus miRNA-5p in saliva may indicate early events of viral reactivation. In this sense, the increased copy numbers of JCPyV-miRNA-5p observed in plasma samples after transplantation, differently from that observed in saliva, could be indicative of potential viral reactivation in another site of the host producing biological fluid with high expression of miRNA-5p due to high viral replication [41,50]. However, the clinical implications of the plasma increased expression observed for JCPyV-miRNA-5p, that it is not the main polyomavirus considered as a risk factor for polyomavirus-associated disease in renal transplantation, at the moment remain to be understood.

This study has some limitations, such as a low number of participants, lack of polyomavirus DNA detection and use of standard stem-loop RT-PCR miRNA assay only to monitor the expression of polyomavirus miRNA-5p. However, previous studies have used this technique and showed that the status of polyomavirus miRNA-5p was more prevalent in the saliva of HIV patients and healthy subjects than that observed for polyomavirus DNA [38]. These results suggested that the high positivity for polyomavirus miRNA-5p in saliva can plays a potential auto-regulation role in the polyomavirus replication in these subjects by reducing the presence of polyomavirus DNA in the oral cavity [38]. Of note, studies investigating the presence or absence of BKPyV, JCPyV, and MCPyV DNA in the saliva or oropharyngeal washes collected from immunocompromised patients and healthy subjects confirm the possibility of a low-replicative form of polyomavirus in the oral cavity [24–28,51,52]. Thus, the reported variation in the status of JCPyV-miRNA-5p in the saliva shortly after transplantation may be implicated in the subsequent polyomavirus reactivation, which can occur in the host after extended immunosuppression and might be associated with the development of polyomavirus-associated diseases.

Finally, several studies have reported that polyomaviruses are potential risk factors for tonsillar tissue, salivary gland, pathogenesis, and pharyngolaryngeal and tongue carcinomas [53–55]. Thus, our study suggests that further investigation on the detection of polyomavirus miRNA-5p in saliva as a noninvasive screening test should be conducted to assess the potential early events associated with polyomavirus reactivation after transplantation.

## Supplementary Material

Supplemental Material
