# Supplementary material for: Detection of polyomavirus microRNA-5p expression in saliva shortly after kidney transplantation
Source: J Oral Microbiol. 2021 Mar 12;13(1):1898838. doi: 10.1080/20002297.2021.1898838 (PMC7971308; doi:10.1080/20002297.2021.1898838)
Supplement: Supplemental Material [file ZJOM_A_1898838_SM7908.docx]

Supplementary data

Table 1s. Immunosuppressive treatment

| Patient ID | Tracolimus (mg Dose/day) | Cyclosporine (mg Dose/day) | Mycophenolate sodium (mg Dose/day) | Azhatioprine(mg Dose/day) | Predinisone (mg Dose/day) | Everolimus  (mg Dose/day) |  | Immunosuppressiveregimen |
| --- | --- | --- | --- | --- | --- | --- | --- | --- |
| 1 | 10 | none | 720 | none | 20 | none |  | Tac + Myf + Pred |
| 4 | 2 | none | none | none | 20 | 6 |  | Tac + Eve + Pred |
| 6 | 20 | none | 1440 | none | 5 | none |  | Tac + Myf + Pred |
| 7 | 6 | none | none | none | 5 | 8 |  | Tac + Eve + Pred |
| 9 | 4 | none | none | none | 30 | 6 |  | Tac + Eve + Pred |
| 10 | 20 | none | none | 175 | 20 | none |  | Tac + Azt + Pred |
| 11 | 8 | none | none | none | 25 | 3 |  | Tac + Eve + Pred |
| 12 | 4 | none | none | 125 | 20 | none |  | Tac + Azt + Pred |
| 13 | none | 250 | none | 100 | 20 | none |  | Cyc + Azt + Pred |
| 14 | 10 | none | none | 125 | 20 | none |  | Tac + Azt + Pred |
| 15 | 20 | none | none | 150 | 30 | none |  | Tac + Azt + Pred |
| 16 | 4 | none | none | none | 20 | 3 |  | Tac + Eve + Pred |
| 18 | 16 | none | none | 125 | 20 | none |  | Tac + Azt + Pred |
| 19 | 16 | none | 720 | none | 20 | none |  | Tac + Myf + Pred |
| 20 | 4 | none | none | none | 15 | 2.5 |  | Tac + Eve + Pred |
| 21 | none | 350 | none | 100 | 15 | none |  | Cyc + Azt + Pred |
| 23 | 12 | none | 1440 | none | 15 | none |  | Tac + Myf + Pred |
| 24 | 12 | none | none | 125 | 30 | none |  | Tac + Azt + Pred |
| 25 | 6 | none | none | none | 20 | 8 |  | Tac + Eve + Pred |
| 29 | none | 300 | none | 100 | 20 | none |  | Cyc + Azt + Pred |
| 30 | 4 | none | none | none | 20 | 3 |  | Tac + Eve + Pred |
| 31 | 12 | none | none | 125 | 20 | none |  | Tac + Azt + Pred |
| 32 | 4 | none | 1440 | none | 20 | none |  | Tac + Myf + Pred |

Tac, tracolumus; Cyc, ciclosporine; Myf, Micophenolatesodium; Azt,Azhatioprine; Pred, Predinisone; Eve, Everolimus

-

Table 2s. Creatinine levels and oral status in study patients

| Patient  ID | Creatinine (mg/dL) | |  | | | Oralhealth status | |  | Orallesions | | |  |  |
| --- | --- | --- | --- | --- | --- | --- | --- | --- | --- | --- | --- | --- | --- |
|  | Before | After | |  | Before | | After |  | Before | After | |  |  |
| 1 | 8.4 | 1.4 | |  | C | | P+C |  | None | Candidiasis | |  |  |
| 4 | 8.6 | 1.2 | |  | N | | P |  | None | Ulceration | |  |  |
| 6 | 12.2 | 1.5 | |  | P | | P |  | None | Ulceration | |  |  |
| 7 | 11.7 | 1.6 | |  | P | | P+C |  | None | Ulceration | |  |  |
| 9 | 4.2 | 1.4 | |  | N | | P |  | None | None | |  |  |
| 10 | 6.7 | 1.5 | |  | P+C | | P+C |  | None | None | |  |  |
| 11 | 5.9 | 1.1 | |  | N | | N |  | None | Ulceration | |  |  |
| 12 | 10.8 | 1.6 | |  | C | | P |  | Candidiasis | None | |  |  |
| 13 | 6.7 | 1.2 | |  | C | | N |  | None | None | |  |  |
| 14 | 6.9 | 1.6 | |  | N | | P |  | None | None | |  |  |
| 15 | 8.7 | 1.8 | |  | N | | N |  | None | None | |  |  |
| 16 | 7.6 | 1.7 | |  | N | | N |  | None | None | |  |  |
| 18 | 14.9 | 0.9 | |  | C | | P |  | None | None | |  |  |
| 19 | 10.1 | 1.9 | |  | N | | N |  | None | None | |  |  |
| 20 | 10.2 | 1.4 | |  | N | | N |  | None | Ulceration | |  |  |
| 21 | 7.3 | 1.1 | |  | C | | P |  | None | None | |  |  |
| 23 | 12.3 | 1.7 | |  | N | | N |  | None | Ulceration | |  |  |
| 24 | 9.3 | 5.7 | |  | N | | P |  | None | None | |  |  |
| 25 | 12.4 | 0.9 | |  | N | | N |  | None | None | |  |  |
| 29 | 10.2 | 1.2 | |  | N | | P |  | None | None | |  |  |
| 30 | 8.5 | 1.3 | |  | N | | P |  | None | None | |  |  |
| 31 | 15.1 | 1.0 | |  | P+C | | P+C |  | None | None | |  |  |
| 32 | 10.2 | 0.9 | |  | C | | P+C |  | None | None | |  |  |
| Maen+ SD | 9.5 + 2.7 | 1.5 + 0.9 | | Total | 10 | | 15 |  | 1 | 7* |  |  |  |

Before, within 24 hours of renal transplantation; After, 60 days after renal transplantation; C, caries;

P,periodontitis; *N, none. * p< 0.05* χ2 test.

Table 3s. Saliva PyV-miRNA-5p expression

| ID Sample | MiRNA-5p copies / ml Saliva | | | | | | | | | | |  |
| --- | --- | --- | --- | --- | --- | --- | --- | --- | --- | --- | --- | --- |
|  | JCPyV | | | BKPyV | | | MCPyV | | | | |  |
|  | B | | A | | B | A | | B | | A | | |
| 1 | | 23040 | - | | 14400 | 18000 | | - | | | - |  |
| 4 | | 14000 | - | | 30000 | 20250 | | - | | | - |  |
| 6 | | 20900 | - | | 38500 | 18000 | | - | | | - |  |
| 7 | | - | - | | 6500 | - | | - | | | - |  |
| 9 | | 17550 | - | | 9000 | - | | - | | | - |  |
| 10 | | 19200 | - | | 28000 | 10000 | | - | | | - |  |
| 11 | | - | - | | 14400 | 11250 | | - | | | - |  |
| 12 | | 422400 | - | | 14000 | - | | - | | | - |  |
| 13 | | - | - | | 22500 | 8000 | | - | | | - |  |
| 14 | | 22000 | 17600 | | - | 22000 | | - | | | - |  |
| 15 | | 35000 | 6500 | | 20000 | 16000 | | - | | | - |  |
| 16 | | - | 5600 | | 28000 | 25000 | | - | | | - |  |
| 18 | | - | - | | 2115 | - | | - | | | - |  |
| 19 | | 16200 | - | | 27000 | - | | - | | | - |  |
| 20 | | 24800 | - | | 18800 | 31500 | | - | | | - |  |
| 21 | | - | - | | 27200 | 24000 | | - | | | - |  |
| 23 | | 5520 | - | | 20700 | 23500 | | - | | | - |  |
| 24 | | - | - | | 4000 | 3400 | | - | | | - |  |
| 25 | | - | 17760 | | 21120 | 22000 | | - | | | - |  |
| 29 | | - | - | | 3500 | - | | - | | | - |  |
| 30 | | - | - | | 20000 | - | | - | | | - |  |
| 31 | | 9500 | 40500 | | - | 4500 | | - | | | - |  |
| 32 | | 23400 | 6000 | | 22500 | 20000 | | - | | | - |  |
| *Total* | | 13 (56%) | 6 (26%)* | | 21 (91%) | 16 (69%) | | 0 | | | 0 |  |
| *Means+ SD* | | 50270+112050 | 15660+ 13443 | | 18677+9737 | 17337+ 7918 | | | - | | - |  |

B and A, before (within 24 hours) and after (after 60 days) renal-transplantation. -, microRNA-5p copies

numbers under the limit of detection of the assay. * *p*<0.05 χ2 test.

Table 4s. Plasma PyV-miRNA-5p expression

| ID Sample | MiRNA-5p copies / ml Plasma | | | | | | | | |
| --- | --- | --- | --- | --- | --- | --- | --- | --- | --- |
|  | JCPyV | | | | BKPyV | | | MCPyV | |
|  | | B | A | | | B | A | B | A |
| 1 | | - | | - | | 3000 | 3000 | - | - |
| 4 | | - | | - | | - | 21000 | - | - |
| 6 | | - | | - | | - | - | - | - |
| 7 | | - | | - | | - | - | - | - |
| 9 | | - | | - | | - | - | - | - |
| 10 | | - | | - | | - | - | - | - |
| 11 | | - | | - | | - | - | - | - |
| 12 | | - | | - | | - | - | - | - |
| 13 | | - | | - | | - | - | - | - |
| 14 | | - | | - | | 20400 | - | - | - |
| 15 | | - | | - | | - | - | - | - |
| 16 | | - | | 60000 | | - | 14000 | - | - |
| 18 | | - | | - | | - | - | - | - |
| 19 | | - | | - | | 12000 | - | - | - |
| 20 | | - | | - | | - | - | - | - |
| 21 | | - | | - | | - | - | - | - |
| 23 | | - | | - | | 10000 | 22000 | - | - |
| 24 | | 2500 | | - | | 12500 | - | - | - |
| 25 | | - | | - | | - | - | - | - |
| 29 | | - | | - | | - | 16000 | - | - |
| 30 | | - | | - | | - | 4000 | - | - |
| 31 | | - | | 1700 | | 21280 | 17860 | - | - |
| 32 | | 13800 | | - | | 3000 | 13500 | - | - |
| Total | | 2 (9%) | | 2 (9%) | | 7 (30%) | 8 (35%) | 0 | 0 |
| Means+ SD | | 8150+7990 | | 30850+41224 | | 11740+7330 | 13920+7102 | - | - |

B and A, before (within 24 hours) and after (after 60 days) renal-transplantation. -, microRNA-5p copies

numbers under the detection limit of the assay.
